# Electromagnetic Analysis of Vertical Resistive Memory with a Sub-nm Thick Electrode

**DOI:** 10.3390/nano10091634

**Published:** 2020-08-20

**Authors:** Batyrbek Alimkhanuly, Sanghoek Kim, Lok-won Kim, Seunghyun Lee

**Affiliations:** 1 Department of Electronic Engineering, Kyung Hee University, Yongin City, Gyeonggi-do 17104, Korea; batyrbek.alimkhan@khu.ac.kr (B.A.); sanghoek@khu.ac.kr (S.K.); 2 Department of Computer Science, Kyung Hee University, Yongin City, Gyeonggi-do 17104, Korea

**Keywords:** memory, VRRAM, sub-nm thin electrode, graphene, EM analysis, device modeling

## Abstract

Resistive random access memories (RRAMs) are a type of resistive memory with two metal electrodes and a semi-insulating switching material in-between. As the persistent technology node downscaling continues in transistor technologies, RRAM designers also face similar device scaling challenges in simple cross-point arrays. For this reason, a cost-effective 3D vertical RRAM (VRRAM) structure which requires a single pivotal lithography step is attracting significant attention from both the scientific community and the industry. Integrating an extremely thin plane electrode to such a structure is a difficult but necessary step to enable high memory density. In addition, experimentally verifying and modeling such devices is an important step to designing RRAM arrays with a high noise margin, low resistive-capacitive (RC) delays, and stable switching characteristics. In this work, we conducted an electromagnetic analysis on a 3D vertical RRAM with atomically thin graphene electrodes and compared it with the conventional metal electrode. Based on the experimental device measurement results, we derived a theoretical basis and models for each VRRAM design that can be further utilized in the estimation of graphene-based 3D memory at the circuit and architecture levels. We concluded that a 71% increase in electromagnetic field strength was observed in a 0.3 nm thick graphene electrode when compared to a 5 nm thick metal electrode. Such an increase in the field led to much lower energy consumption and fluctuation range during RRAM switching. Due to unique graphene properties resulting in improved programming behavior, the graphene-based VRRAM can be a strong candidate for stacked storage devices in new memory computing platforms.

## 1. Introduction

With the progress in the field of deep learning and neural networks, the concept of computation is altering toward a data-centric paradigm [1,2]. Computationally intensive operations required in such applications also demand complex memory platforms. Recent advances in non-volatile memory technology such as Flash were primarily focused on multilevel storage capability and transistor size reduction. However, inherent drawbacks such as an increased bit-error rate and reliability degradation accompany such technology node downscaling [3,4].

Emerging memory devices with potentially higher performance and memory density compared to conventional devices can become an effective solution in today’s post-Moore era [5,6,7]. These technologies use conceptually different mechanisms of storing data, which are generally independent of the node size. Resistive random access memory (RRAM) is one of such devices with high potential for next-generation storage class memory [8]. Furthermore, due to its intrinsic high density, endurance, and fast programming requiring small energy consumption, RRAM is a promising candidate for data-intensive tasks widely used in brain-inspired computing [9,10,11]. With its simple metal–insulator–metal structure, extreme scalability and Complementary Metal–Oxide–Semiconductor (CMOS) compatibility are also RRAM’s important attributes [12].

Functioning as both storage cell and electronic synapse, RRAMs can be integrated as a cross-point array, where the active memory layer is sandwiched between a word-line and a bit-line. Such a structure allows us to address every cell in the array, and importantly, to perform vector–matrix multiplication (VMM) operation, a critical step in neuromorphic computing [13,14]. Both the downscaling of the feature size and the increase in interconnect resistance are expected to cause array response deterioration, which will significantly degrade the overall performance of the memory platform [14,15].

One method to alleviate the highlighted problem and achieve higher density is to stack several layers of cross-point arrays to form a 3D structure [16]. An improved version of such structure is a vertical RRAM (VRRAM) array where memory cells are inserted between multilayer plane electrodes and vertical pillar electrodes. A VRRAM array offers higher bit-cost efficiency compared to a simple stacked array because VRRAMs require only one pivotal lithography step [17,18,19]. Several works concerning the vertical downscaling of VRRAMs and adopting atomically thin graphene electrodes have been introduced for both stand-alone (1R) and built-in selector (1S1R) structures [20,21,22]. These devices are known to have lower switching voltages and currents compared to devices fabricated with conventional metal electrodes. However, an in-depth analysis of such a device’s electromagnetic characteristics and a fundamental explanation of its low energy consumption does not exist. In this work, we focus on experimental verification and electromagnetic characterization of VRRAMs composed of either graphene or conventional metal electrodes and compare the results. Theoretical background and a compact model are also derived. The obtained results are especially useful for both the research community and device designers for further implementation of sub-nm thick materials in memristors and other neuromorphic components.

## 2. Materials and Methods

### 2.1. Fabrication

Fabrication of the graphene-based VRRAM can be understood as serial stages of graphene transfer, dry etching to form a trench, deposition of active memory layer, and a pillar electrode. Graphene (monolayer on copper foil, Graphene Supermarket) was transferred to the top of the Al_2_O_3_ (5 nm) dielectric (atomic layer deposition) of the deposited SiO_2_(1000 Å)/Si substrate. Metal contact pads Ti/Pt were deposited and patterned by evaporation and lift-off processes. The SiO_2_ (60 nm) isolation layer was deposited using the Low Pressure Chemical Vapor Deposition (LPCVD) method, followed by a deep trench forming using dry etching. Next, an active memory layer, HfO_2_ (5 nm), and a pillar electrode, TiN (200 nm), were sequentially fabricated using Atomic Layer Deposition (ALD) and DC sputtering, respectively. Finally, a metal contact opening was conducted by dry etching. More detailed information regarding graphene transfer and fabrication of multiple layers of plane electrodes in 3D VRRAMs can be found in previous works [20,22,23]. The active device area, i.e., the contact area of the metal oxide and the edge of the plane electrode, was found to be 0.5 μm^2^ and 0.03 μm^2^ for the VRRAM with platinum and graphene electrodes, respectively.

### 2.2. Device Characterization and Modeling

For high-resolution transmission electron microscopy (HR-TEM) imaging, a Tecnai TF-20 Field Emission Gun/TEM (FEI Company, UK) functioning at 200 kV was used. For the *I-V* voltage sweep measurement, a Keithley 4200-SCS parameter analyzer with an integrated remote pre-amplifier (Keithley, OH, USA), a Keithley 707B Switch Matrix, and a Keysight 81150A arbitrary signal function generator (Keysight, CA, USA) were used. The compliance currents (I_CC_) for the conventional metal (Pt) and graphene-based VRRAMs were 30 μA during SET with a maximum sweep of 2.5 V, and 5 μA with a maximum voltage sweep of 1 V, respectively. The modeling of the device response was performed in Verilog-A language, and the simulation was compiled in Simulation Program with Integrated Circuit Emphasis (HSPICE, I-2013.12-SP1 64-BIT, Synopsys Inc., Mountain View, CA, USA). In addition, computational analysis was conducted in MATLAB (R2017a, MathWorks, MA, USA).

## 3. Results and Discussion

**Figure 1:**
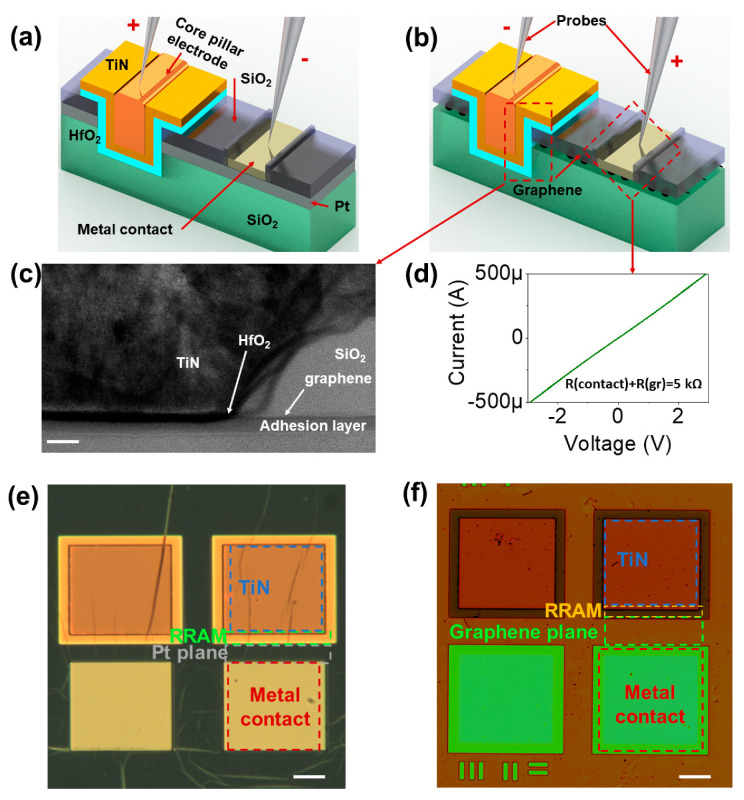
The structure of the single device RRAM in 3D vertical architecture. (**a**,**b**) The schematic of the VRRAM with conventional metal (Pt) and 2D graphene edge electrodes, respectively. The probes are configured for SET programming operations; (**c**) high-resolution TEM image of the cross-sectional view of the graphene-based VRRAM (Scale bar: 10 nm); (**d**) current response of the sole monolayer graphene sheet with no active memory layer; (**e**,**f**) microscopic image (top view) of the Pt- and graphene-based VRRAMs (Scale bar: 20 μm).

Figure 1a,b shows the schematic of the fabricated VRRAM with conventional metal (Pt) and graphene plane electrodes. The active memory layer is sandwiched between the pillar and edge plane electrodes, satisfying the vertical structure. It should be noted that specified layouts demonstrate the structure of the single cell of the vertical resistive switching memory with built-in metal contacts for electrical characterization. The probes contacting metal pads and pillar electrodes in both devices are configured to perform SET programming operations.

The thickness of the Pt electrode of the control device is 5 nm, whereas the graphene monolayer is known to be 3 Å thin [20,23,24]. For reference, in this study, the pillar (TiN) and plane (Pt or graphene) electrodes are presented as top and bottom electrodes, unless otherwise stated. Figure 1c depicts a high-resolution TEM image of the cross-sectional view of the VRRAM with the graphene electrode. Here, a graphene sheet was accurately transferred on top of the adhesion layer (see Materials and Methods section). Moreover, given the proper etching aspect ratio that is practiced by the industries, the resulted trench is expected to be principally abrupt. It is known that graphene has exclusive thermal and electrical properties suitable for electronic applications [25]. Therefore, the current response of the memory-free VRRAM is as shown in Figure 1d, indicating the expedient conductivity of the graphene material, including specific semi-metal/metal contact. Microscopic images of the fabricated VRRAMs with metal (control sample) and graphene electrodes are shown in Figure 1e,f.

**Figure 2:**
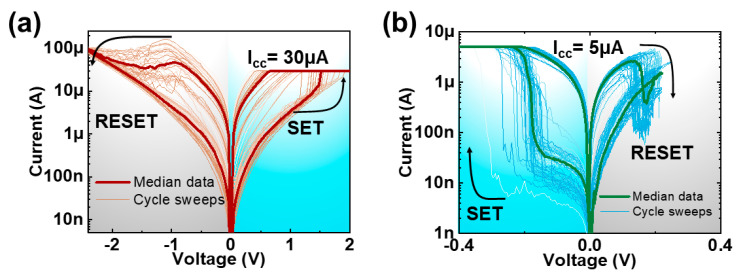
*I-V* response of the (**a**) Pt- and (**b**) graphene-based VRRAMs with multiple DC cycles. Blue and gray regions represent SET and RESET operations in each device, respectively.

The experimentally obtained *I-V* response of multiple cycles of the VRRAM with Pt and graphene electrodes are depicted in Figure 2a,b. Blue represents the SET operation region, i.e., transition from high (HRS) to low (LRS) resistance states, whereas gray reflects the RESET region, switching from LRS to HRS. It is important to note that programming voltages is substantially smaller in the graphene-based VRRAM (V_SET_ = −0.2 V; V_RESET_ = 0.2 V) when compared to the memory with the Pt electrode (V_SET_ = 1.4 V; V_RESET_ = −2.3 V), providing ultra-low power switching. In addition, resistive switching occurs in different polarities of the voltage sweep. This implies that in conventional VRRAMs, the SET is obtained by biasing the pillar electrode with positive amplitude voltage and grounding the metal plane electrode (Figure 1a). In contrast, for the graphene-based VRRAM, one can expect the SET by applying a positive voltage on the graphene plane and grounding pillar electrodes. Furthermore, the switching current for the conventional metal-based VRRAM is 30 μA for SET and 100 μA (self-compliance) for RESET. Integrating the graphene as a plane electrode will provide 5 μA for both programming configurations. Initially, the current compliance of 5 μA, similar to the one utilized in the programming operations in graphene-based memory, was used during the forming process. In the case of the VRRAM with a metal electrode, the impact of the current compliance levels, including 30 μA used during the experimental forming/measurements, on the resistance distribution will be discussed later in this study. In addition, there is considerable variability in the *I-V* response of both devices, and it is important to note that such stochasticity in the operation is usually understood as an intrinsic phenomenon of filamentary-type RRAM devices. In general, the temporal and spatial stochasticity in filamentary memory devices, including the studied VRRAMs with graphene and metal plane electrodes, is mostly related to the low probability of reproducing the exact the same number of nanoparticles (oxygen vacancies) and shape of conductive filaments in each consecutive programming cycle and device, respectively [26]. Additionally, the presence of the defects accommodated in the insulator that trap the charging electrons also contributes to the variability of the devices.

**Figure 3:**
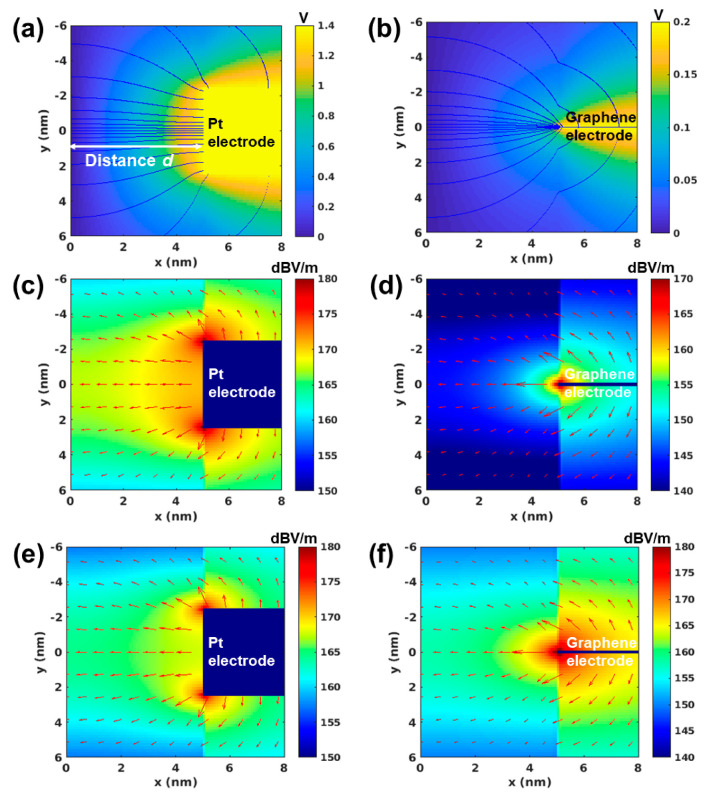
A comparison of the electric potential for (**a**) Pt and (**b**) graphene electrodes, and the electric field for (**c**) Pt and (**d**) graphene electrodes. Similarly to the experimental characterization settings, 1.4 V of electric potential is applied to the Pt electrode and 0.2 V to the graphene electrode while the TiN electrode is grounded; (**e**,**f**) The electric field strength comparison under equal potential conditions (1V) for graphene and Pt-based VRRAMs. The TiN electrode is placed at the plane of x = 0 (nm) and plane electrodes are sandwiched between the SiO_2_ dielectric layers. The figures (**a**) and (**b**) show the electric potentials with the electric flux lines overlaid. In (**c**–**f**), the strength of the electric field is denoted by the colormap in the unit of (dBV/m). The red arrows also represent the electric field distribution including the direction of the field.

For the electromagnetic analysis, we assume that Pt and graphene electrodes are placed apart from the TiN electrode by a distance *d* (Figure 3). The thickness of the Pt electrode (5 nm) is much larger than the graphene thickness (3 Å). When a programming voltage is applied on Pt and graphene electrodes while the TiN electrode is grounded, we compute and compare the electric field appearing in the dielectric. It is well known that in a static condition, the electric field ***E*** is expressed as the gradient of the electric potential ***V***:

(1) E=−∇V

From Gauss’s law, the static electric potential satisfies the Laplacian equation in a source-free region:

(2) $$\nabla^{2}V=0$$

The electric potential is subject to the boundary condition, which enforces that the potential is zero (grounded) for the TiN electrode (x = 0 nm), while the Pt or graphene electrode has the applied voltage according to the programming settings. There are many methods to solve the Laplacian equation with the boundary condition, such as the finite element method [27] or method of moments [28]. Among those, in this work, the variational method [29] is chosen to solve the field distribution due to its simplicity in implementation. According to the variational method, solving the Laplacian equation is equivalent to the problem that minimizes an integral *U* defined as

(3) $$U=\frac{\varepsilon }{2}\int {\left(\nabla V\right)}^{2}dv=\frac{\varepsilon }{2}{\int \left(\frac{dV}{dx}\right)}^{2}+{\left(\frac{dV}{dy}\right)}^{2}dv$$

subject to the same boundary condition imposed on the potential, where ε is the permittivity of the dielectric memory cell between the electrodes. It consists of high dielectric medium HfO_2_ (ε_r_ = 22) in the region of (*x < d*) and SiO_2_ (ε_r_ = 3.7) in the other region (*x* > *d*) (Figure 3a–f). In other words, the variational method has converted the Laplacian equation into an optimization problem. The 2-dimensional function that minimizes the objective function (3) is the electric potential that satisfies the Laplacian Equation (2). Since the objective function (3) that we aim to minimize has a convex property, it can be solved via convex optimization [30]. Lastly, the electric field is obtained from the negative gradient of the electric potential.

Figure 3a–d demonstrates the applied potentials with electric flux overlaid and electric field distributions for Pt and graphene electrodes under programming conditions used during the experimental characterization. Thus, the peak electric field of the graphene-based under 0.2 V applied potential is 169 dBV/m, which is slightly less than the peak strength at the edge of the Pt electrode (180 dBV/m) under the significantly higher potential of 1.4 V. Moreover, for the applied unit voltage difference (1 V; Figure 3e,f), it can be seen clearly that the peak strength of the electric field (179.0 dBV/m; 8.9 × 10^8^ V/m) for a thin graphene electrode increased by approximately 71% when compared to that of the thick Pt electrode (174.3 dBV/m; 5.2 × 10^8^ V/m). A stronger electric field from the thinner and sharper graphene electrode causes the required voltage for the formation of the conductive filament with a firm core to be lowered in the dielectric memory cell. It is also worth noting that the thick Pt electrode has a peak electric field near the top and bottom edge of the electrode. This indicates that the conductive filaments (formed by oxygen vacancies) tend to form at the top or bottom corner of the Pt electrode, and this may lead to variances of switching characteristics since there are two polar fields.

**Figure 4:**
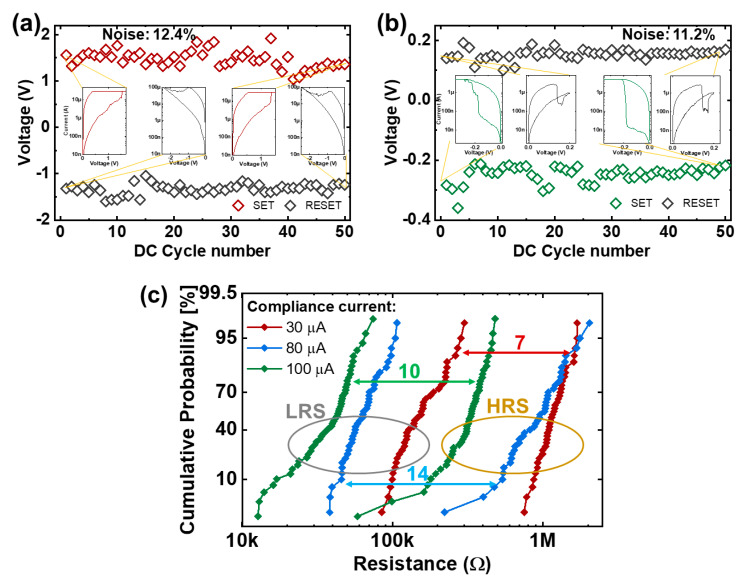
Programming voltage fluctuation for (**a**) Pt- and (**b**) graphene-based VRRAMs. Inset: *I-V* characteristic of the 1st and last sweep for SET and RESET. (**c**) Cumulative probability of the resistance distribution in the VRRAM with a conventional metal electrode programmed at various compliance currents.

Figure 4a,b demonstrates the programming voltage variations for the SET and RESET operations in multiple consecutive DC sweeps for both devices. The obtained data can be useful in injecting the random noise for the single device model. Notably, the programming variation in the graphene-based memory is marginally less (11.2%) than the VRRAM with Pt electrode (12.4%), which can be related to the small switching current and the focused location of the peak electric field and its strength. In addition to the fact that graphene provides VRRAM to operate at considerably smaller voltage/current ranges, there is a favorable tradeoff between the programming noise and memory window. However, this does not apply for conventional metal-based RRAM, since a minor reduction in programming noise will require memory to work at depressed current ranges. This can be achieved by artificially setting a lower current compliance, which, in turn, leads to the undesirable memory window shrink. Since high current compliance will lead to the formation of the conductive filament (CF) with a greater area, more traps can be accommodated indicating the dominance of the random telegraph noise (RTN) [31]. As a result, at the depressed current range, an evident tradeoff between variability and memory window size is expected. Figure 4c demonstrates the cumulative probability of the resistance distribution in the VRRAM with conventional metal plane electrodes programmed at various compliance currents. It is worth noting that both the variability and memory window were reduced for the memory with less compliance current.

**Figure 5:**
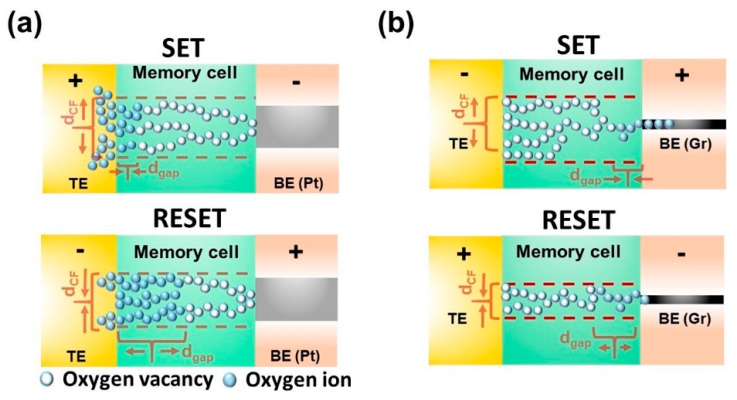
The illustration of the modeling of conductive filament evolution during SET/RESET programming operations in the VRRAM with (**a**) Pt and (**b**) graphene edge electrodes.

The development of the conductive filament (CF) can be described by two spatial means, including vertical and horizontal growth induced by the electric field [15,32,33]. Conventionally, in a 2D cross-point RRAM structure, the vertical growth refers to the tunneling gap, whereas the horizontal increase denotes the radius of the CF; however, these notions are interchanged in the 3D vertical structure due to the peculiar location of the top (pillar) and bottom (plane) electrodes, as shown in Figure 5. It is worth noting that the unique nature of programming polarity (Figure 1b) and ultra-low switching power (Figure 2b) are highly related to the graphene edge electrode and its interface performing as an oxygen reservoir [20]. Contrastingly, in the conventional VRRAM structure, the TiN pillar electrode, particularly the TiO_x_N_1–x_ barrier layer at the TiN/HfO_2_ interface, operates as an oxygen reservoir to collect or discharge oxygen ions consuming considerably large energy [17,20]. This is because conductive filament formation and rupture are associated with oxygen migration to and from the TiN/HfO_x_ interface, respectively, expedited by the electric field and Joule heating. It should be noted that in the graphene-based VRRAM, there is a slight current increase during the RESET operation (Figure 2b), which can be related to its double structure with two potential active electrodes. This means that alternating SET and RESET operations cause a small portion of the oxygen to be stored in the TiN/HfOx interface as residual oxygen ions. Thus, during the RESET operation, these residual oxygen ions migrate towards the opposite direction, causing a slight current increase under the DC voltage sweep condition. However, it is important to note that the activation energy of the oxygen ion diffusion in graphene (≈0.7 eV) is considerably lower than in TiN (≈2.1 eV) [34,35]. This implies that for the programming conditions of the graphene-based RRAM, including vastly low current compliance, SET and RESET voltages are not sufficient enough to store a greater portion of the oxygen ions in the TiN electrode, indicating stable operation of the graphene-based memory. In addition, the stochastic behavior of the RRAM devices can be explained by the random location of oxygen vacancies around the CF body [32]. There is an exponential dependence on the tunneling gap of the oxide material and linear dependence on the width of the CF [33].

In 3D vertical memory cell, the horizontal evolution of the conduction path can be modeled as the generation or rupture of the CF. The Stanford RRAM model accurately demonstrates the RRAM resistance switching process based on the tunneling gap distance variation [36,37,38,39].

(4) $$I=I_{0}\times \exp(\frac{-d_{gap}}{d_{0}})\times \sinh(\frac{V}{V_{0}})$$

where *d_gap_* is the tunneling gap distance and *V* is the applied voltage. Several tunneling mechanisms may occur in the RRAM memory cell, where the majority demonstrates the exponential dependence of the current density on the applied electric field and tunneling distance. This may include Fowler–Nordheim (FN) field emission, direct tunneling, trap-assisted tunneling (TAT), or Poole–Frenkel emission.

The vertical evolution of the conduction path can be described as an increase or decrease in the width of the CF. In its turn, the geometrical shape of the CF can be modeled as a cylinder or truncated cone. As a result due to the dominant Ohmic nature of the conduction path, there is a linear dependence of current density on the diameter of the CF [15,33].

(5) $$I=\pi d_{CF}^{2}\cdot V/(4\rho \cdot t_{OX})$$

where *d_CF_* is the diameter and *ρ* is the resistivity of the CF, and *t_OX_* is the oxide (memory cell) thickness. Thus, the model shown in (5) depicts RRAM device resistance switching relying on diameter evolution. It should be noted that both the evolution rate of tunneling gap (*d_gap_*) and the diameter (*d_CF_*) of the CF are related to the probability of the oxygen ions to obtain enough energy to exceed the energy barrier (*E_a_*) following the Arrhenius law [40].

(6) $$\frac{d\langle d_{gap}\rangle }{dt}=-v_{0}\times \exp(-\frac{E_{A}}{kT})\times \sinh(\gamma \times \frac{a_{0}}{t_{OX}}\times \frac{qV}{kT})$$

(7) $$\gamma =\gamma_{0}-\beta \times d_{gap}^{\alpha }$$

(8) $$\frac{d\langle d_{CF}\rangle }{dt}=-K\cdot v_{0}\times \exp(-\frac{E_{A}}{kT})\times \sinh(\xi \times \frac{qV}{kT})$$

where *a*_0_ is the ion hopping distance and *γ* is a local field enhancement factor that regards distinct characteristics for tunneling gap-based bipolar (SET/RESET) switching in oxide material [41,42]. In contrast, *ξ* in (8) is a field dependence factor which accounts for the decrease in the electric field effect after the formation of the CF due to radial diffusion of oxygen ions induced by the local temperature and Coulomb repulsion (attraction) [15,33,43]. *K*, *v*_0_, *α*, *γ*_0_, *β* are parameters that have a considerable effect on the *I-V* curve and can be extracted upon experiment results [41].

By applying the time integral, the evolution of both tunneling gap and diameter can be determined in (9) and (10), respectively. Furthermore, the effect of the thermal noise can be evaluated given that the variation is estimated to a tolerable level.

(9) $${d_{gap}|}_{t+\Delta t}=\int \left(\frac{d\langle d_{gap}\rangle }{dt}+N_{t}(0,\sigma (T))\right)dt$$

(10) $${d_{CF}|}_{t+\Delta t}=\int \left(\frac{d\langle d_{CF}\rangle }{dt}+N_{t}(0,\sigma (T))\right)dt$$

**Figure 6:**
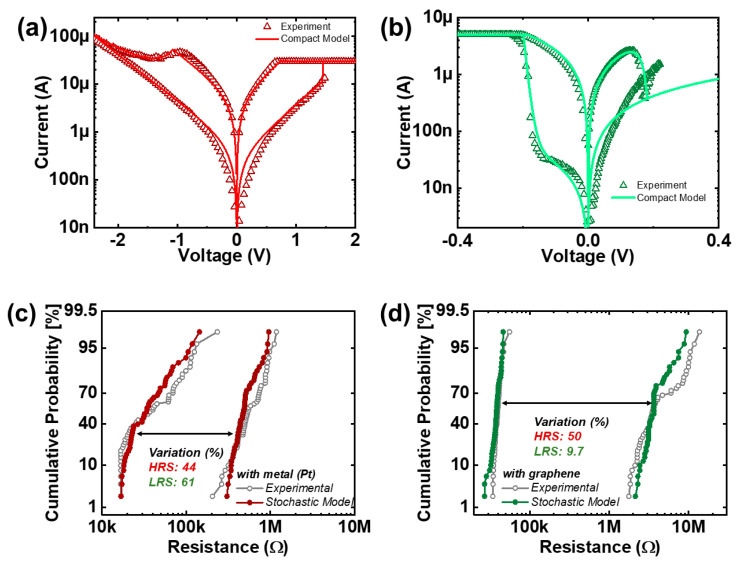
Experimentally verified Verilog-A compact model fitting of the VRRAM with (**a**) Pt and (**b**) graphene electrodes; (**c**,**d**) The resistance distribution from the experimental measurements and models with an injected stochastic feature for the Pt- and graphene-based VRRAM, respectively.

For the effective implementation of a compact model for the VRRAM with metal and graphene electrodes, some notions should be considered. In virtue of the dominant switching process based on the increase/decrease in the horizontal length of the CF in the VRRAM with the Pt electrode (Figure 5a), the Verilog-A model can be simplified to focus on the evolution of the tunneling gap. In the graphene-based VRRAM, as there is lack of an interfacial layer (oxygen reservoir) in the TiN/HfO_x_ interface and the thickness of the graphene plane electrode is substantially smaller in comparison with the active pillar electrode, for reasons of simplicity, the switching process in the Verilog-A model may prevalently depend on the radial evolution of the CF (Figure 5b). Consequently, the *I-V* characteristics of the VRRAM device model with conventional metal and graphene edge electrodes are as shown in Figure 6a,b. Furthermore, the derived model inherently has a statistical aspect, and during the experimental measurement, it was noted that both device responses have a specific variability common to filamentary-type RRAMs. The temporal variations shown in Figure 4a,b can be further injected into the model as the random noise in the field enhancement and dependence factors leading to the variability of the programming voltages. In addition, the read noise, predominantly caused by the RTN, is extracted from the experimental results and can be modeled as the fluctuation of the conductive filament diameter/tunneling gap and the current read. Figure 6c,d demonstrates the resistance distribution from the experimental result and the model with injected stochasticity. The simulated models are in good tolerance with experimental data; thus, it is believed that the obtained results will be useful in the estimation of sub-nm graphene material in neuromorphic computing applications.

## 4. Conclusions

In summary, highly dense memory can be achieved by integrating a sub-nm thick plane electrode such as graphene in a 3D VRRAM structure. It was found that VRRAMs with such a sub-nm thick electrode exhibited a very different device response compared to that of a conventional metal electrode, resulting in low programming voltages and power consumption. The electromagnetic analysis showed that the electric field strength was increased in a 0.3 nm thick plane electrode by 3.7 × 10^8^ V/m, accounting for approximately a 71% increase. In addition, the relatively thick Pt electrode has a peak electric field near the top and bottom edges, causing the formation of the branched CFs at two polar fields, which may lead to fluctuations during the resistance switching operations. On the other hand, the peak electric field strength is concentrated at the center of the edge for a sub-nm thick electrode. Based on the highlighted notions and experimental measurements, we derived a theoretical basis of the switching process of the VRRAM with conventional metal and graphene electrodes. As a result, the models that include the intrinsic responses of both devices were extracted. It is believed that the obtained results will be highly useful in the estimation of the potential of the graphene-based 3D memory at the circuit and architecture levels for designing in-memory processing and neuromorphic computing units.
